# Time-frequency transformation integrated with a lightweight convolutional neural network for detection of myocardial infarction

**DOI:** 10.1186/s12880-024-01502-2

**Published:** 2024-12-02

**Authors:** Kashvi Ankitbhai Sheth, Charvi Upreti, Manas Ranjan Prusty, Sandeep Kumar Satapathy, Shruti Mishra, Sung-Bae Cho

**Affiliations:** 1 School of Computer Science and Engineering, Vellore Institute of Technology, Chennai, 600127 India; 2 Centre for Cyber-Physical Systems, Vellore Institute of Technology, Chennai, 600127 India; 3 Department of Computer Science, Yonsei University, Seodaemun, Seoul, 03722 South Korea; 4 Centre for Advanced Data Science, Vellore Institute of Technology, Chennai, Tamil Nadu 600127 India

**Keywords:** Electrocardiograms (ECGs), ECG signals, Time-frequency transformation, Discrete wavelet transform (DWT), Convolutional neural networks (CNN), Myocardial infarction (MI)

## Abstract

Myocardial infarction (MI) is a life-threatening medical condition that necessitates both timely and precise diagnosis. The enhancement of automated method to detect MI diseases from Normal patients can play a crucial role in healthcare. This paper presents a novel approach that utilizes the Discrete Wavelet Transform (DWT) for the detection of myocardial signals. The DWT is employed to break down ECG signals into distinct frequency components and subsequently to selectively filter out noise by thresholding the high-frequency details, resulting in denoised ECG signals for myocardial signal detection. These denoised signals are fed into lightweight one-dimensional Convolutional Neural Networks (CNN) for binary classification into Myocardial Infarction (MI) and Normal categories. The paper explores three distinct approaches: utilizing all signals, incorporating under-sampling and up-sampling to address class imbalances, with both noised and denoised signals. Evaluation of the suggested model is done with the help of two publicly available datasets: PTB-XL, a large publicly available electrocardiography dataset and PTB Diagnostic ECG Database. Results obtained through 5-fold cross-validation on the trained model show that the model has achieved an accuracy of 96%, precision of 97% and F1 score of 95%. On cross-validation with the PTB-ECG dataset, this paper achieved an accuracy of 91.18%.

## Introduction

The 2023 World Health Report published by the World Heart Federation, reveals that cardiovascular diseases (CVDs) continue to be a leading global cause of mortality globally. In 2021, CVDs caused about 20.5 million deaths, making up around one-third of all deaths worldwide [1]. The identification of cardiovascular diseases, is often facilitated through the utilization of electrocardiograms (ECGs), a non-invasive and indispensable diagnostic tool. The model detailed in this research accepts the ECG signal as its input and subsequently conducts binary classification to determine the presence of Myocardial Infarction (MI) or normal cardiac conditions.

In cardiovascular disease diagnosis, the 12-lead ECG has traditionally relied on manual interpretation by experienced cardiologists, a process that is both time-consuming and requires significant skill [2]. Yet, this approach poses inherent challenges, including the potential for human error, thereby risking incorrect clinical decisions and endangering patient health. Given the rapid advancements in ECG technology and the persistent shortage of cardiologists, the pursuit of precise and automated ECG signal diagnosis has emerged as a compelling research avenue for scientists.

Over the past decade, researchers have undertaken numerous initiatives aimed at unlocking the diagnostic potential of 12-lead clinical ECGs. These efforts have predominantly leveraged publicly available, large-scale ECG data repositories. The existing body of literature on ECG databases reveals a dual methodological approach: signal processing and machine learning. In the field of diagnosing cardiovascular diseases, deep neural networks have become a crucial factor. These sophisticated learning models have proven effective in improving the precision of cardiovascular disease diagnoses by analysing ECG signals [3]. Utilizing a series of varied neural network layers for the gradual extraction of higher-level features, these networks iteratively enhance the underlying structure on which they are constructed. Across various fields where artificial intelligence algorithms find application, deep neural networks are currently at the forefront of innovation.

The research study primarily focused on enhancing the accuracy of MI detection within the broader context of coronary artery disease (CAD), which is a significant subset of cardiovascular disease (CVD) [4]. As a crucial component of this research paper, PTB XL dataset [5] was used to specifically target the detection of MI and Normal (NORM) cardiac conditions. The PTB XL dataset served as a valuable resource due to its extensive and comprehensive collection of ECG signals, making it well-suited for the study’s primary objective of classifying these two important cardiac conditions.

In this paper a pioneering approach to MI detection, which harnesses the capabilities of 1D Convolutional Neural Networks (CNN) while integrating the Discrete Wavelet Transform (DWT) as a novel denoising mechanism has been introduced. The primary contribution of this research lies in the development of a custom-designed 1D CNN model tailored for this specific diagnostic task. This novel architecture not only effectively learns discriminative features from ECG signals but also demonstrates its robustness in the presence of noise and artifacts. By integrating the DWT for signal denoising, the quality of ECG data is significantly enhanced, thus enhancing the model’s capability to extract meaningful patterns associated with MI. The necessity for DWT as a denoising tool, rather than just a feature extraction method, is crucial as it helps remove noise and unwanted artifacts from the ECG signals, which are often present due to electrical interference or patient movement. This ensures that the model is trained on cleaner, more accurate data, leading to improved diagnostic accuracy and more reliable detection of MI events. Furthermore, this paper explores three distinct approaches for MI detection: involving the classification of all signals into MI and Normal categories, leveraging undersampling and upsampling to address class imbalances, with both noised and denoised signals. This versatility ensures the adaptability of the model to various real-world scenarios, underlining its practical utility. The outcomes of this research represent a significant step forward in the realm of automated MI diagnosis, with the potential to impact the field of cardiology and healthcare by improving early detection and, consequently, patient outcomes. The significant contributions of this research include:

1. The incorporation of the DWT as a denoising technique for ECG signals.
2. The development of a custom-designed lightweight 1D CNN model tailored for MI detection.
3. Utilization of undersampling and upsampling as an innovative technique to address class imbalances, enhancing the practical applicability and fairness of the model.
4. Investigation of different hyperparameter tuning methodologies, aiming to optimize the performance of the proposed model.

The paper follows this structure: firstly, it covers the related work in the initial section, followed by Section “Datasets – PTB XL and PTB ECG” providing an overview of the dataset. Section 4 describes the proposed methodology. Additionally, Section “Performance measures” explores various performance measures used to evaluate classifier performance. Section “Results and discussions” presents results and discussion illustrating classifier performance. Finally, Section “Conclusion” concludes the paper.

## Related works

Research [3] showed that a CNN with added entropy features outperformed both CNN and SincNet architectures, achieving the highest accuracy rates for various classification tasks, including 2, 5, and 20 classes with rates of 89.2%, 76.5%, and 69.8%, respectively. However, the paper observed limitations related to overfitting in their models. In the study [6], various deep learning techniques are applied to distinguish normal and abnormal ECG in the MIT-BIH arrhythmia database. The best accuracy achieved 83.4%, was obtained with five-fold validation with CNN-LTSM. A notable advantage of this proposed paper is operation without the need for noise filtering or feature engineering. The study [7] aimed to devise an automatic acute MI detection method employing CNN and Long Short-Term Memory (LSTM) on echocardiography data. The approach yielded an overall classification accuracy of 85.1% for the left ventricular long-axis view and 83.2% for the short-axis view. However, the study is limited to acute anteroseptal infarction and does not cover other coronary-dominant regions.

The utilization of deep learning is fully incorporated in [8], where a CNN neural network model is extensively trained and applied to examine ECG abnormality recognition and prediction. The model achieves an accuracy of 86% in recognizing ECG abnormalities. The paper [9] achieved accuracy of 87.78% with CNN architecture to distinguish between 17 classes of MIT-BIH Arrhythmia database. The paper proposed a deep neural network model for cardiac arrhythmia and includes a self-supervised approach for ECG beat signal prediction. In the study [10], an algorithm for MI detection directly from ECG data is proposed. The approach uses CNN, to classify ECG data and discern the presence of MI. The CNN model achieved an impressive accuracy of 87% when applied to the Physikalisch-Technische Bundesanstalt database.

The paper [11] introduces an approach for automated ECG identification and classification. It employs Deep Convolutional Neural Network (DCNN) with Bidirectional Long Short-Term Memory (BiLSTM) to extract ECG features. Using the 2017 PhysioNet/CINC challenge dataset it achieved an accuracy of 89.3%. However, as noted in the paper, the filtering algorithm works well on this dataset but is less effective with data from different ECG devices. The research [12] focuses on ECG classification employing 1D convolutional neural networks combined with FCN layers on pre-processed time-series data, achieving a notable validation accuracy of approximately 86%. An advantage of the method is the ability to classify ECG data from unstructured, unbalanced 1D time series, making it useful when medical specialists are unavailable for feature engineering.

In research [13] Enhanced Deep Neural Network (EDN) model comprising of CNN and LSTM techniques was proposed for classification of MI using the PTB Diagnostic ECG database. It achieved an accuracy of 88.89%. The paper [14] proposes a method to classify MI using multi-lead ECG signals. They transformed ECG signals into a density model and derived the feature vector through hidden Markov models (HMMs). The classification of MI was accomplished using Gaussian mixture models (GMMs), resulting in an accuracy of 82.50%. Along with ECG classification log-likelihood value was also calculated serving as statistical feature for each heartbeat’s ECG complex.

The study [15] proposes a multilabel classification method for ECG recordings into five cardiac states using data from PTB-XL (100 Hz down-sampled version). ECG signals are converted into natural visibility graphs, with features extracted from the first few diagonals of the adjacency matrix and node weights. ResNet and Inception models are applied for classification, achieving 89.71% accuracy, 79.61% F-score, and 93.46% AUC. However, the visibility graph induction faces computational limitations due to the adjacency matrix’s size, especially with longer time series, requiring down sampling or fixed-length time window processing.

The paper [16] applies a sliding window approach without overlap to segment beats. The beats are processed through a CNN and attention layer, followed by Bi-LSTM and rhythm-level attention. This approach achieved a macro-averaged ROC-AUC of 0.9216, mean accuracy of 88.85%, and a maximum F1 score of 0.8057 on the PTB-XL dataset, with the highest class-wise accuracy (91.58%) achieved for the HYP class. The paper [17] classifies the PTB Diagnostic dataset with an accuracy of 88.33%, sensitivity of 89.47%, and specificity of 87.80%. It uses a symlet scaling filter and denoising process for preprocessing, followed by AlexNet, a CNN model, to extract deep features, and then applies ELM for classification.

## Datasets – PTB XL and PTB ECG

The analysis presented in this study relies on data sourced from the PTB-XL (PhysioNet/PTB-XL) database, which is a highly valuable resource offering access to a wide array of 12-lead ECG waveforms [18]. This extensive dataset comprises a remarkable collection of 21,799 records collected from 18,869 unique patients, each with a 10-second ECG record. One of the remarkable features of the PTB-XL dataset is its inclusivity, encompassing individuals spanning a wide age range, from infants to adults over 95 years of age, with a mean age of 62. This age diversity ensures that the dataset provides a comprehensive and lifelong snapshot of ECG patterns, which is crucial for understanding cardiac health over the human lifespan [4]. Furthermore, PTB-XL dataset includes a substantial number of healthy ECG records, amounting to 9,514 samples. This abundance of healthy records makes it a valuable resource for the study of normal cardiac rhythms and serves as an invaluable reference for baseline ECG patterns. In addition to the healthy records, the dataset al.so contains a significant number of ECG records associated with MI, with a total of 5,469 samples available for analysis.

**Fig. 1:**
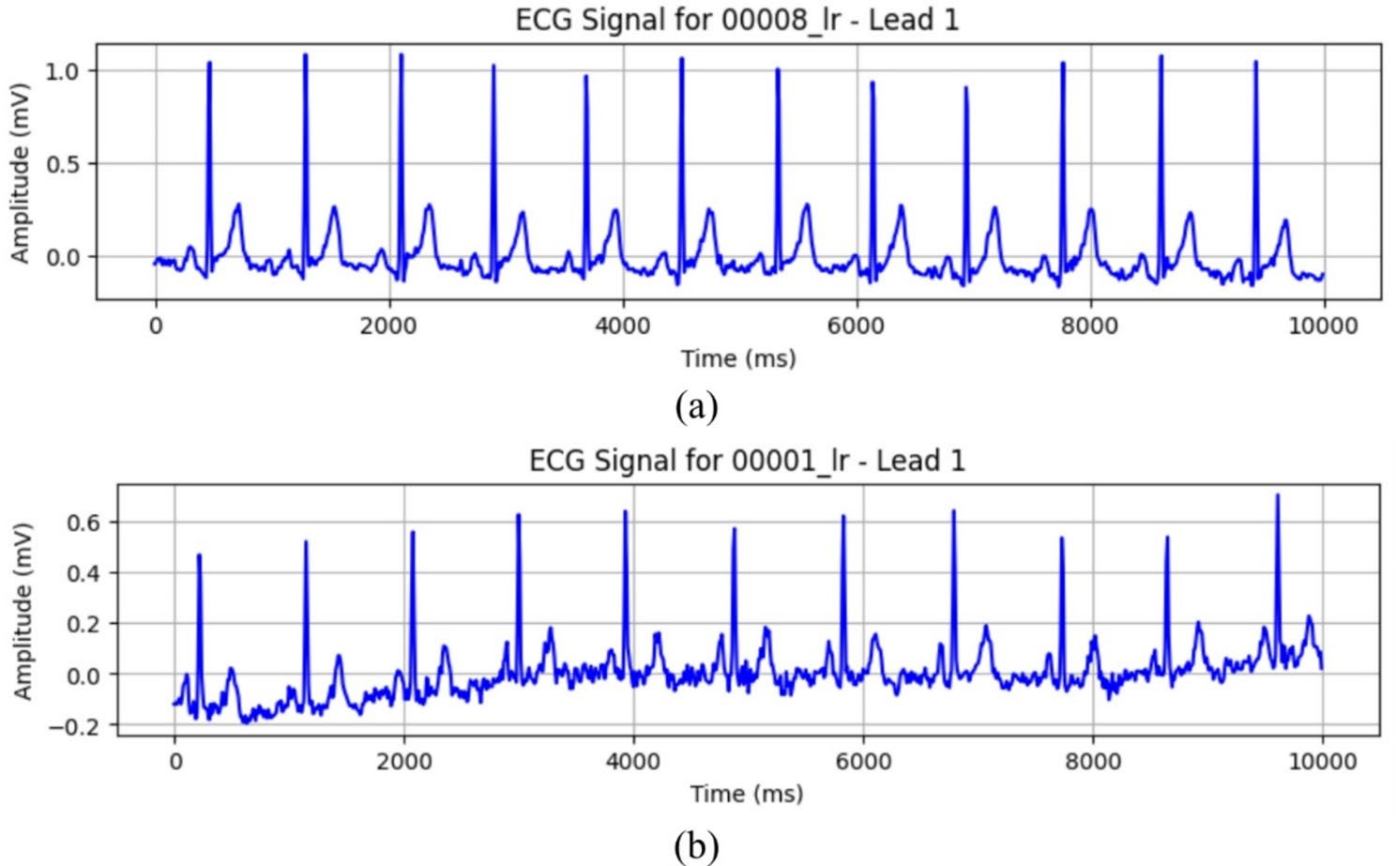
(**a**) ECG signal representing myocardial infraction; (**b**) ECG signal representing normal

The PTB-XL dataset consists of paired .hea and .dat files for each patient. These files contain essential information about the ECG recordings and the actual signal data. In the context of this dataset, Normal (NORM) ECG signals exhibit characteristic patterns associated with healthy cardiac activity, while MI signals represent distinct electrical patterns related to this critical cardiac condition. Figure 1 illustrate how these ECG signals typically appear.

The other dataset used is PTB (Physikalisch-Technische Bundesanstalt) ECG database [19]. It comprises a vast collection of high-quality ECG recordings obtained from a diverse population, making it an essential dataset for various cardiac studies. The database features 549 records, encompassing both healthy subjects and patients with a variety of cardiac conditions, ensuring a wide range of ECG patterns for comprehensive analysis. These recordings are sampled at 1000 Hz, enabling detailed examinations of cardiac electrical activity. Notably, it comprises 148 MI records and 52 healthy control records. Each record is accompanied by a comprehensive set of annotations, providing crucial information on various ECG events and abnormalities, facilitating precise diagnostic and research efforts.

**Fig. 2:**
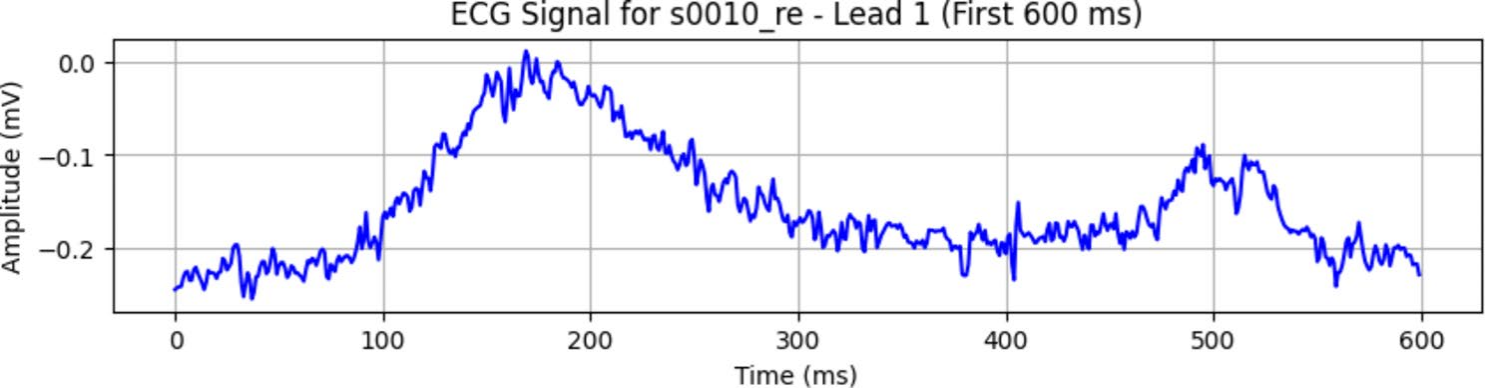
ECG signal in PTB ECG database

Moreover, the PTB ECG database offers a remarkable diversity of ECG signals, including data from exercise tests, 24-hour ECG recordings, and standard 12-lead ECGs, making it an invaluable resource for a multitude of applications. This dataset’s extensive clinical information, diversity of cardiac conditions, and high-quality ECG recordings render it a fundamental component of this research, enabling the development and validation of a robust diagnostic model for MI detection. Figure 2 illustrate how these ECG signals typically appear in PTB-ECG database.

## Proposed methodology

**Fig. 3:**
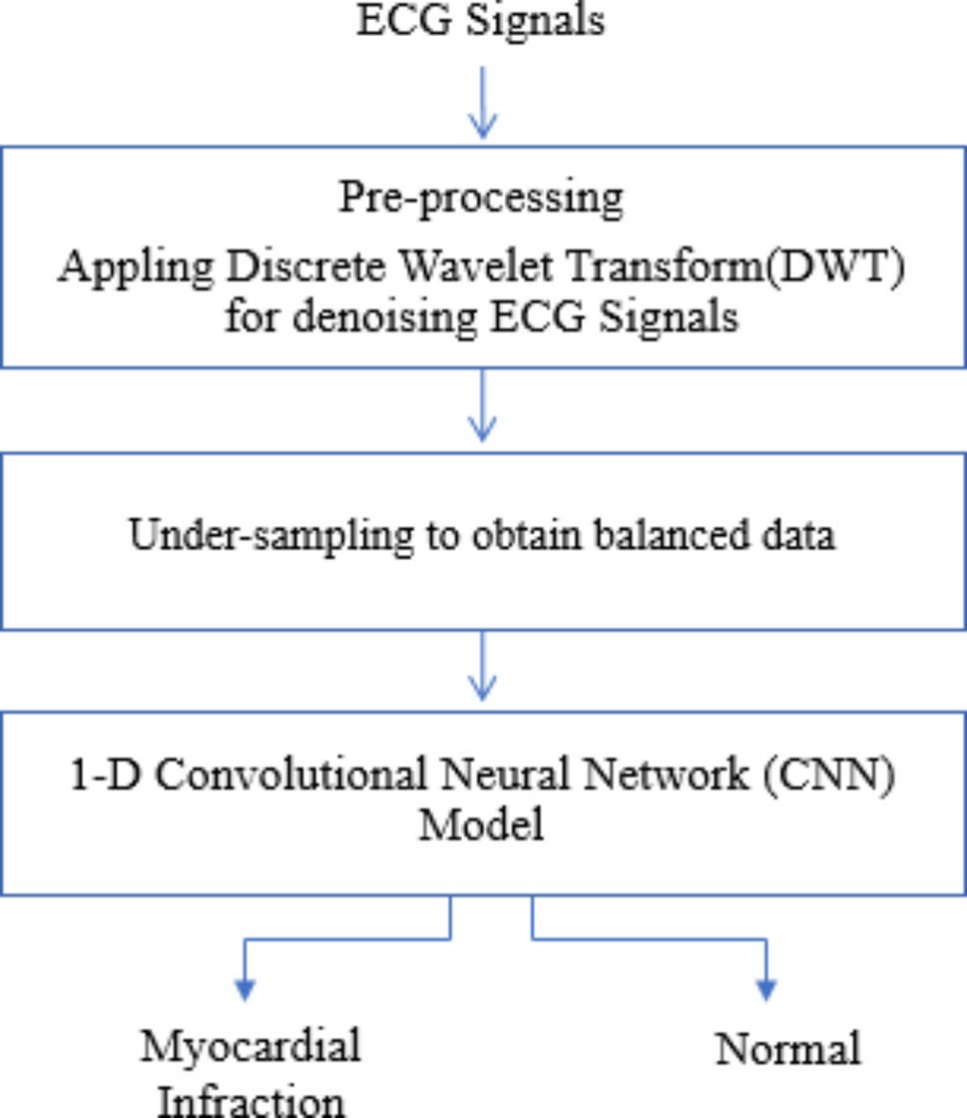
Block diagram of the suggested model

In the proposed architecture, the foundation is laid by obtaining critical ECG data, an indispensable resource for cardiovascular research and diagnosis. The raw ECG data was sourced from the PTB-XL database [18], consisting of continuous ECG signals sampled at 100 Hz. In the proposed approach for ECG signal classification, the proposed method begins with pre-processing the raw ECG signals to remove noise and artifacts, ensuring data quality. To enhance denoising capabilities, the DWT is employed, a powerful technique for extracting relevant features and reducing noise in the signals. To address class imbalance issues, under-sampling is applied to the dataset to ensure balanced representation across classes in the training data. For the classification stage, a lightweight 1D CNN model is constructed, optimized for efficiency in terms of computational and memory resources. This choice of a 1D CNN architecture allows for effective pattern recognition within the ECG signals, specifically by capturing localized dependencies, which are crucial for accurate classification tasks. In initial tests, a 2D CNN was considered, but the increased computational demands and longer processing times led to selecting the more efficient 1D CNN (See Fig. 3).

### Discrete wavelet transform (DWT)

**Fig. 4:**
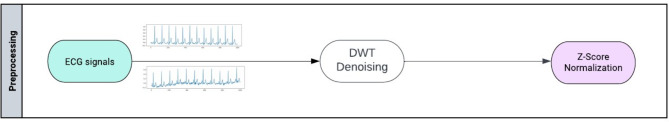
Preprocessing stage involving DWT for denoising

In the realm of ECG signal processing, the DWT plays a pivotal role in the quest for denoising and feature extraction. The aim of this section is to elucidate the underlying methodology and principles of DWT, specifically its application in preprocessing ECG signals for classification and analysis. Figure 4 represents the diagram of pre-processing.

The PyWavelets (pywt) library [20] was as harnessed as a fundamental tool in this process, which facilitated the intricate steps of DWT, thresholding, and signal reconstruction. The initial step in the preprocessing pipeline involved the one-dimensional decomposition of the ECG signal using the wavedec function, a fundamental operation in DWT. The decomposition breaks down the original signal into its constituent wavelet coefficients, revealing the signal’s frequency content at different scales. Mathematically, the DWT can be expressed as:

1 $$\:DWT\left(x\right)=\sum\:\left(x\left[n\right]*\psi\:[n-k]\right)$$

Where, DWT(x) represents the wavelet coefficients, x[n] denotes the original signal values, ψ[n - k] is the wavelet function, and k denotes the translation parameter. The decomposition provides a multi-resolution representation of the signal, offering insights into both low and high-frequency components.

Subsequent to decomposition, a critical step in DWT-based denoising is the application of a threshold. This operation, performed on the wavelet coefficients, selectively removes high-frequency noise while preserving significant signal features. The choice of PyWavelets enabled the application of thresholding with ease, enhancing the denoising process. Mathematically, the thresholding operation can be described as:

2 $$\:Thresholded\_coefficients\left[n\right]\:=\:f\left(coefficients\right[n],\:threshold)$$

Here, Thresholded_coefficients[n] are the modified coefficients, coefficients[n] represent the original wavelet coefficients, and f(coefficients[n], threshold) is a thresholding function that determines whether a coefficient should be retained or set to zero. After thresholding, the final step entailed signal reconstruction using the waverec function, which combined the thresholded coefficients to generate a denoised ECG signal. This reconstructed signal retained the vital information of the original ECG while removing undesirable noise artifacts. The signals were then filtered to isolate the recordings classified as ‘NORM’ – ‘Healthy’ and ‘MI’ – Myocardial Infraction. In order to standardize the data for analysis, the Z-score normalization method was applied [21]. Where, x is an individual data point, x̅ is mean and σ is standard deviation.

3 $$\:Z\:score=\:\frac{x-\bar{x}}{\sigma\:}$$

### Under-sampling and up-sampling

In this study, the focus was on the classification of ECG signals, a critical task for medical diagnosis and monitoring. To tackle the imbalance between the “NORM” (Normal ECG) and “MI” (Myocardial Infarction) classes, a random under sampling technique was implemented on the training data. The dataset originally contained 9514 instances of normal ECG signals and 5469 instances of MI cases. To equalize the representation of these two classes, the majority class was randomly under sampled, which in this case was the “NORM” class. This ensured that each class had the same number of instances, preventing the classification model from being biased toward the majority class. By under sampling the majority class, the potential risk of the model favouring class imbalance was mitigated and achieved a more balanced training set. Up-sampling the training data was also experimented as a method to address the data imbalance. This involved randomly selecting and duplicating signals within the minority “MI” class to augment its sample size. This paper carefully preserved the diversity of ECG signals by randomly choosing a subset of “NORM” and “MI” instances for training, maintaining the integrity of the original data.

### Lightweight convolutional neural network (CNN) model

**Fig. 5:**
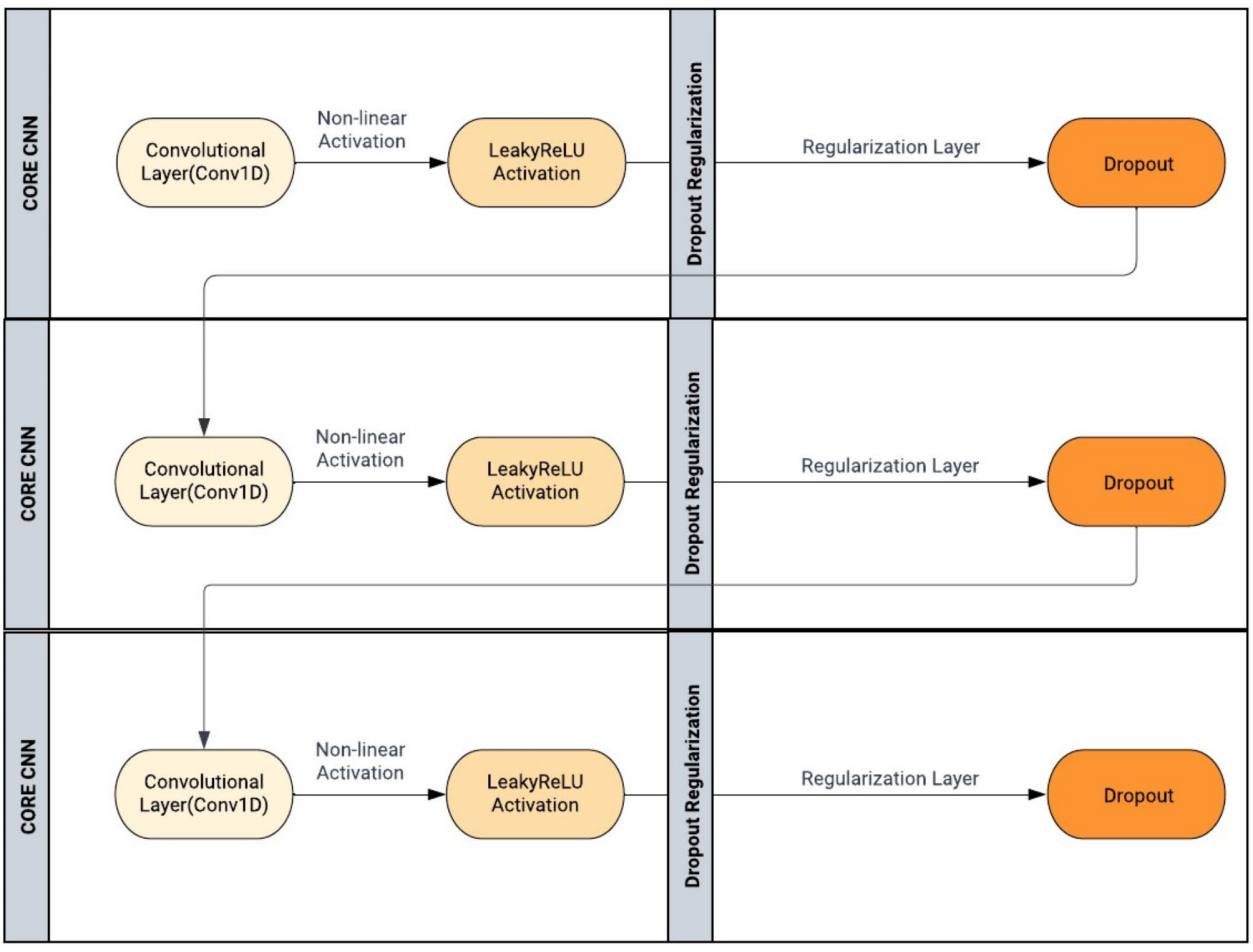
Lightweight CNN architecture used in the proposed model

In the model architecture, a sequence of layers to process the ECG signals has been employed. Three 1D convolution layers were used with varying filter sizes to extract relevant features from the ECG signals. Following each convolutional layer, a Leaky ReLU activation function is applied, which allows the network to learn complex patterns and a Dropout layer to prevent overfitting. Core CNN architecture is described in Fig. 5.

The Rectified Linear Unit (ReLU) produces an output of 0 when the input is less than 0, and for input values greater than 0, the output equals the input. The Leaky Rectified Linear Unit, derived from ReLU, incorporates a slight slope for negative values instead of a flat slope. This design prevents complete deactivation of neurons, enabling the network to learn from negative values. This enhancement contributes to the network’s capacity to comprehend intricate patterns within the data. It is defined by the following equation:

4 $$\:f\left(x\right)=\text{m}\text{a}\text{x}(0,x)$$

**Fig. 6:**
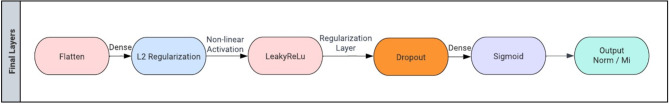
Final layers of the CNN network

Figure 6 describes the layers after the convolution layers. The output was flattened to prepare it for fully connected layers. L2 regularization (also called Ridge regularization) was used. wi represents an individual weight in the neural network and λ (lambda) is the regularization strength or hyperparameter, controlling the trade-off between fitting the training data and minimizing the magnitude of the weights.

5 $$\:L2\left(w\right)=\lambda\:\times\:\sum\:{{w}_{i}}^{2}$$

Finally, Sigmoid layer was used for the binary classification. The sigmoid function (also called logistic function) is an S-shaped curve that transforms the input values into range between 0 and 1. Values nearing 0 suggest a low likelihood, whereas those approaching 1 indicate a high probability., it is considered highly valuable for binary classification problems like the one discussed in this paper. Where, S(x) is sigmoid function and e is Euler’s number.

6 $$\:S\left(x\right)=\:\frac{1}{1+\:{e}^{-x}}$$

**Table 1:** Hyperparameter table of the suggested CNN model

Hyperparameters	Values
MI instances	5469
NORM instances	9514
Learning rate	10^− 3^
Minimum LR	10^− 4^
Batch size	64
Epochs	10
Optimizer	Adaptive moment estimation (Adam)
Loss function	Binary cross-entropy

The performance of the lightweight CNN model was assessed by employing various evaluation metrics, including precision, recall, accuracy, and the F1-score. These metrics were derived from the confusion matrix and allowed comparison of the proposed model with existing ones. Additionally, this paper uses the Area Under the Curve (AUC) score, which is determined from the Receiver Operating Characteristic (ROC) curve, to evaluate the proposed model’s ability to distinguish between positive and negative examples. The AUC score indicates the likelihood that a randomly selected positive example will be ranked higher than a randomly selected negative example. To ensure the robustness of proposed model, two validation techniques were employed: 5-fold cross-validation on our pre-trained model [22] and cross-validation with PTB-ECG dataset [19]. Table 1 is the hyperparameter table of the suggested CNN model.

## Performance measures

The table that summarizes the findings of a classification algorithm is called confusion matrix. This matrix provides the key performance metrics, which include recall, accuracy, precision, and F1-score. Accuracy is the proportion of correct predictions among the entire set of predictions (Eq. 7). Precision represents the positive identifications that were correct (Eq. 8). Recall measures the ratio of correctly predicted positive samples to the total number of positive predictions (Eq. 9). F1 score is calculated as the harmonic mean of precision and recall (Eq. 10). In the following equations, TP stands for true positives, which signifies the accurate identification of positive predictions. TN represents true negatives, indicating correct classification of negative predictions. FP denotes false positives, representing incorrect identification of cases as positive. FN stands for false negatives, indicating the misclassification of cases as negative. K-fold cross-validation is a method employed in machine learning for assessing model performance. The procedure entails partitioning the training data into k folds or subsets. Subsequently, the model is trained on k-1 folds and tested on the remaining fold. This cycle is reiterated k times, ensuring that each fold serves as the test set precisely once. The average of the performance metrics from each iteration is then employed as the model’s performance estimate. In this paper, five-fold cross-validation technique has been implemented [22].

7 $$\:Accuracy=\frac{TN+TP}{TN+TP+FN+FP}$$

8 $$\:Precision=\:\:\:\:\:\frac{TP}{TP+FP}$$

9 $$\:Recall=\frac{TP}{TP+FN}$$

10 $$\:F1\:score=2\times\:\frac{Precision\times\:Recall}{Precision+Recall}$$

## Results and discussions

### Performance analysis

In order to comprehensively assess the effectiveness of the proposed algorithm, the code has been rigorously tested using multiple approaches. These approaches encompass denoised under-sampling, denoised processing on the entire signal, and denoised up-sampling. Additionally, the algorithm was assessed under noise-based under-sampling, noise processing on the entire signal, and noise-based up-sampling scenarios. The diversity of these testing methodologies provides a robust examination of the algorithm’s capabilities under different conditions, enabling a more thorough understanding of its efficacy across various signal processing scenarios. The outcomes from these approaches are delineated in Tables 2 and 3, and 4, presenting the undersampling, all signals, and upsampling results, respectively.

#### Under-sampling results

In the denoised under-sampling tests, the model achieved its highest performance at 60 epochs, with a training accuracy of 0.97 and a test accuracy of 0.83. The precision and recall were both 0.83, and the F1 score was 0.82, indicating balanced performance in identifying the target class while minimizing false positives. The AUC value of 0.90 further emphasizes the model’s strong discriminatory ability, particularly in detecting MI events in the ECG data. As the number of epochs decreased, from 50 to 10 epochs, performance gradually declined, with the test accuracy dropping to 0.84–0.85 and the F1 score dipping slightly. This suggests that longer training periods allow the model to better learn the underlying data patterns, whereas shorter training epochs may result in underfitting, where the model hasn’t fully captured the complexity of the data.

In the noisy under-sampling tests, the model showed reasonable performance with 10 epochs, achieving a training accuracy of 0.87 and a test accuracy of 0.84. The precision, recall, and F1 score were all around 0.83, with an AUC value of 0.92, similar to the denoised case, suggesting the model maintained good discriminatory power even under noise. At 30 epochs, training accuracy improved to 0.926, and test accuracy increased to 0.825, though performance was still slightly lower than in the denoised scenario. The addition of noise in the training data seems to impact the model’s ability to generalize, as reflected in the marginally lower test accuracy and F1 scores when compared to the denoised case. However, the AUC score of 0.91 indicates that the model was still able to distinguish between classes effectively, even with the noise present.

Overall, while the denoised under-sampling approach provided better generalization and higher test accuracy, the model showed a robust performance even when noise was introduced. The denoised data helped achieve the best results, particularly in terms of test accuracy, F1 score, and overall model stability, while the noisy data resulted in a slight degradation of performance metrics, though still offering solid discriminatory power.

**Table 2:** Under-sampling results

Signals	Epochs	Train accuracy	Test accuracy	Precision	Recall	F1 score	AUC
Denoise	10	0.87	0.86	0.85	0.85	0.85	0.92
	20	0.91	0.829	0.83	0.81	0.82	0.90
	30	0.93	0.84	0.83	0.83	0.83	0.90
	50	0.968	0.827	0.82	0.82	0.82	0.91
	60	0.97	0.83	0.83	0.82	0.82	0.90
Noise	10	0.87	0.84	0.83	0.84	0.83	0.92
	30	0.926	0.825	0.82	0.81	0.82	0.91

#### All signals results

On evaluation of the model with all signals, including both denoised and noisy data, the results remain relatively consistent, with test accuracies ranging from 0.84 to 0.87. The model trained on denoised signals at 10 epochs yields a high test accuracy of 0.86, with a precision of 0.87 and a recall of 0.83, leading to an F1 score of 0.84. Interestingly, the test accuracy does not drastically improve with increasing epochs, suggesting that the model may have reached a plateau where additional training epochs contribute less to performance enhancement.

While the precision remains relatively high, the recall shows some variability, dipping slightly as the model is exposed to more noise. This indicates that while the model is effective at identifying positive cases (MI), there might be room for improvement in reducing false negatives. The AUC value consistently hovers around 0.90 to 0.93, demonstrating that the model retains its ability to distinguish between the classes even as it is exposed to more complex signal data.

**Table 3:** All signals results

Signals	Epochs	Train accuracy	Test accuracy	Precision	Recall	F1 score	AUC
Denoise	5	0.84	0.85	0.84	0.95	0.89	0.90
	10	0.89	0.86	0.87	0.83	0.84	0.93
	20	0.89	0.85	0.86	0.81	0.83	0.91
Noise	10	0.89	0.87	0.87	0.85	0.86	0.93
	15	0.89	0.86	0.86	0.83	0.84	0.92
	20	0.91	0.86	0.86	0.83	0.84	0.92

#### Up-sampling results

In the denoised up-sampling tests, the model showed good performance, with a training accuracy of 0.84 and test accuracy of 0.84 at 5 epochs. As the epochs increased, training accuracy reached 0.94 at 20 epochs, while test accuracy remained steady at around 0.85–0.846. Precision, recall, and F1 score stabilized at 0.84 and 0.82–0.83, with AUC consistently at 0.91, indicating strong class differentiation.

In the noisy up-sampling tests, performance was similar but slightly lower. At 5 epochs, test accuracy was 0.83, with precision, recall, and F1 score around 0.83. As training progressed, test accuracy decreased slightly at 20 epochs to 0.837, while AUC remained stable at 0.91. This suggests that while the model benefits from more training, noise impacts its generalization, leading to reduced performance compared to the denoised case.

**Table 4:** Up-sampling results

Signals	Epochs	Train accuracy	Test accuracy	Precision	Recall	F1 score	AUC
Denoise	5	0.84	0.84	0.84	0.83	0.83	0.91
	10	0.89	0.85	0.85	0.84	0.84	0.92
	20	0.94	0.846	0.85	0.82	0.83	0.91
Noise	5	0.8417	0.83	0.82	0.84	0.83	0.91
	10	0.8962	0.85	0.85	0.83	0.84	0.91
	20	0.94	0.837	0.84	0.82	0.82	0.91

Upon reviewing the results across all approaches, it is clear that the model trained with denoised data and under-sampling for 60 epochs yields the best overall performance, with a training accuracy of 0.97 and a test accuracy of 0.83. This model strikes the best balance between reducing noise and avoiding overfitting, and as a result, it has been selected for further evaluation through five-fold cross-validation and validation with the PTB ECG dataset.

In the denoised up-sampling tests, the model showed good performance, with a training accuracy of 0.84 and test accuracy of 0.84 at 5 epochs. As the epochs increased, training accuracy reached 0.94 at 20 epochs, while test accuracy remained steady at around 0.85–0.846. Precision, recall, and F1 score stabilized at 0.84 and 0.82–0.83, with AUC consistently at 0.91, indicating strong class differentiation.

#### Computational time comparison

The decision to adopt a 1D convolutional layer architecture over a 2D convolutional layer was driven by considerations of computational efficiency and compatibility with available system resources. The system configuration comprises a 13th-generation Intel processor operating at 2.40 GHz and 16GB of RAM, which places practical limits on model complexity and training times. The 1D convolutional layer architecture was identified as more lightweight and resource-efficient than the 2D counterpart. Performance benchmarking, presented in Table 5, indicates that training with the 1D Conv layer architecture required an average of 995 s for 5 epochs, whereas the 2D Conv layer took significantly longer, averaging 1428 s for the same number of epochs. This increase in training time with the 2D Conv layer, coupled with the relatively modest system specifications, motivated the selection of the 1D Conv layer as a more optimal choice for this study.

Ultimately, the 1D Conv layer architecture was prioritized due to its suitability for faster processing and lower memory demands, aligning with the need for an efficient yet capable model under limited computational resources. This choice underscores the importance of balancing model complexity with system constraints, particularly when working within environments with restricted hardware capacities.

**Table 5:** Approximated timing for 1D and 2D conv

Epochs	1D (seconds)	2D (seconds)
5	4975	7140
10	9950	14,280
20	19,900	28,560
60	59,700	85,680

**Fig. 7:**
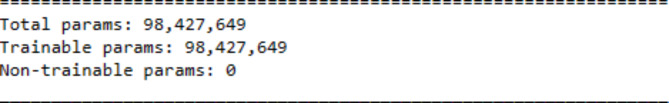
Total parameters

**Fig. 8:**
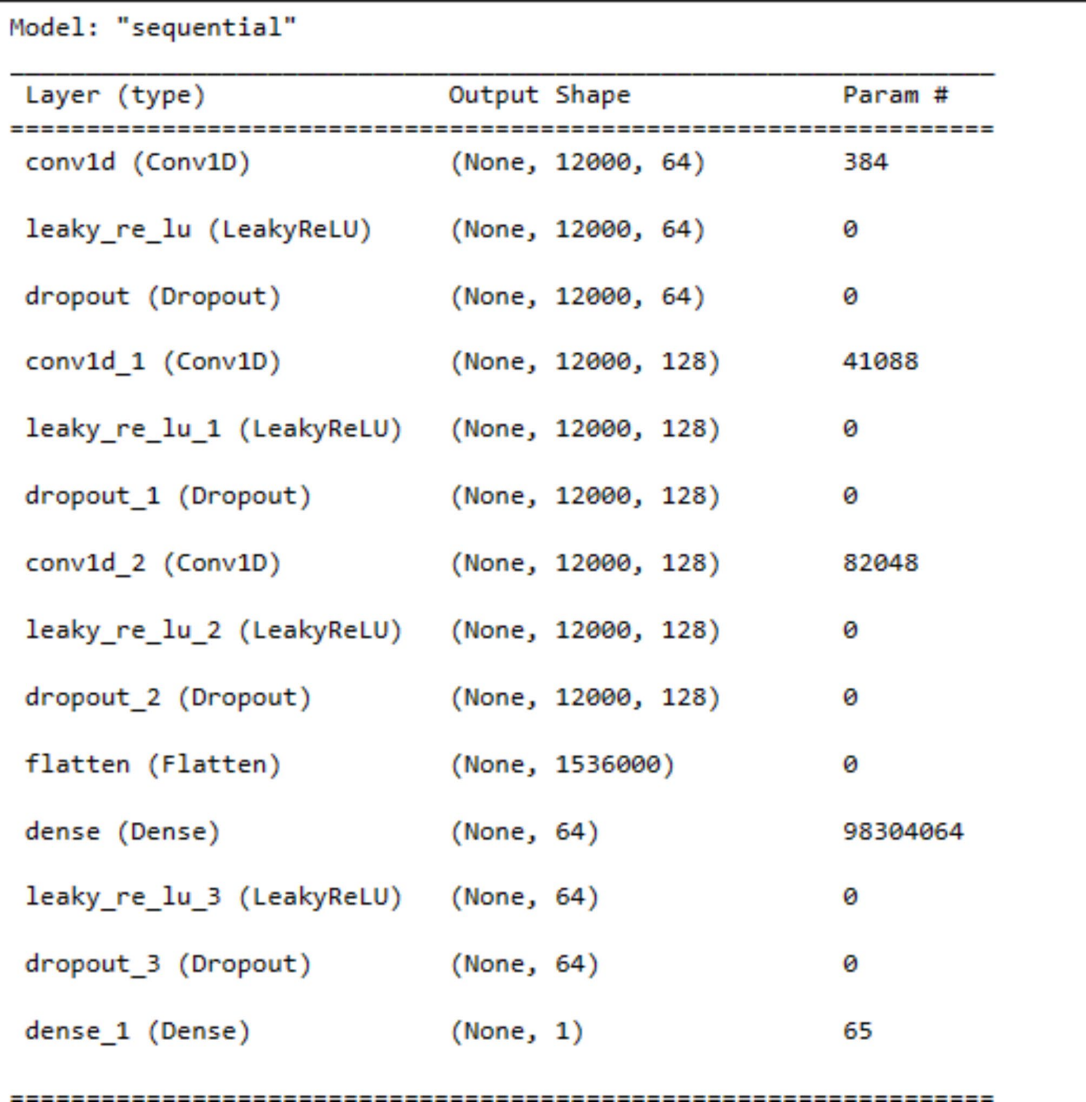
Proposed model summary

Figures 7 and 8 provide additional context for understanding the architecture of the model. Figure 7 illustrates the total parameters involved in the model, highlighting its complexity, while Fig. 8 offers a comprehensive model summary, showcasing the layers and operations used in the neural network architecture.

### Training result analysis

**Fig. 9:**
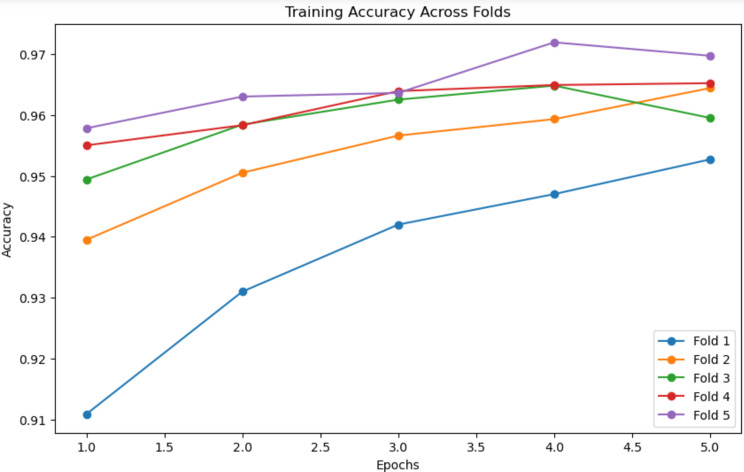
Graph depicting the relationship between accuracy and epochs during training analysis

As illustrated in Fig. 9, the training accuracy demonstrates a clear improvement over the course of the epochs. In Fold 1, the accuracy begins at around 91%, showing that the model is starting from a relatively modest level of performance. As training progresses through the epochs, accuracy gradually increases, reaching 94% by the fifth epoch. This consistent rise suggests that the model is effectively learning the patterns within the data and is refining its parameters with each pass.

The subsequent folds (Fold 2 to Fold 5) exhibit a more pronounced improvement. From the second fold onwards, training accuracy consistently climbs, ultimately reaching a peak of 97% in Fold 5. This trend highlights the model’s ability to adapt and improve as it sees different training data splits, suggesting that using multiple folds helps the model generalize better to unseen data. The steady upward trajectory, particularly across the later folds, demonstrates that with increasing training epochs, the model’s performance stabilizes and its ability to classify ECG signals accurately is enhanced. It is notable that while accuracy continues to rise initially, it starts to plateau towards the later epochs, which indicates that the model is nearing its optimal performance level.

This observation reinforces the idea that a balanced number of epochs is key to maximizing accuracy while preventing overfitting. The improvements observed in the later folds further suggest that cross-validation, which splits the data into different folds for testing, contributes to better robustness and more reliable model evaluation.

### Test result analysis

It was observed that the denoised under-sampled model trained over 60 epochs exhibited the highest training accuracy, as documented in Tables 2 and 3, and 4. Consequently, the under-sampled models were selected for subsequent evaluation. A 5-fold cross-validation was employed for models trained over 10 and 20 epochs, incorporating early stopping [23]. However, for the model achieving the highest accuracy, a 5-fold cross-validation was conducted without early stopping.

Based on the results in Table 6, it is observed that the model’s performance consistently improved as the number of epochs increased, with test accuracy, precision, recall, F1 score, and Area Under the ROC Curve (AUC) all rising. This trend indicates that the model benefits from longer training durations, leading to better generalization and more accurate predictions. The model trained over 60 epochs demonstrates the highest performance across all metrics, showing the best test accuracy, precision, recall, F1 score, and AUC during the 5-fold cross-validation evaluation.

**Fig. 10:**
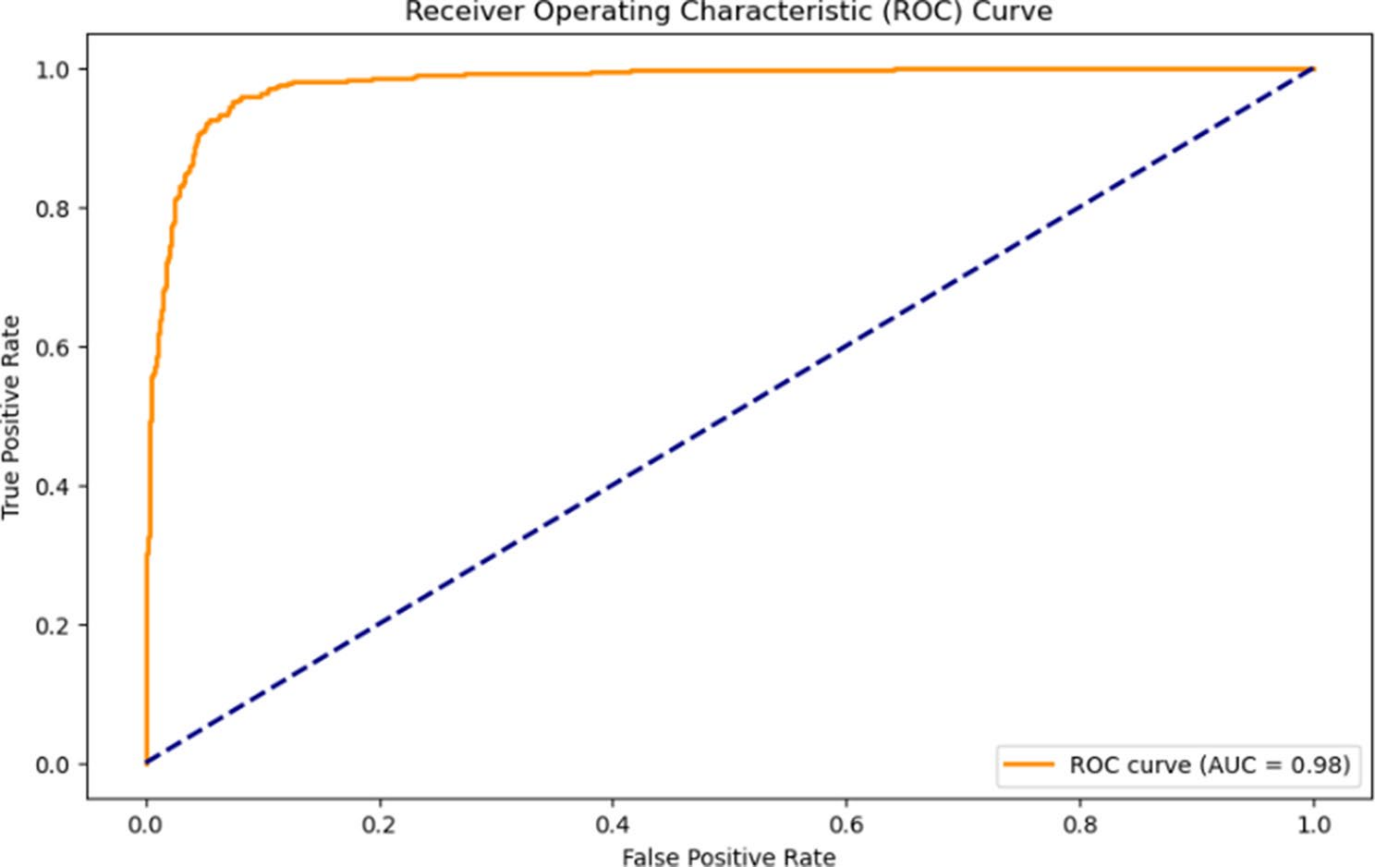
ROC curve for the suggested model

Figure 10 shows the Receiver operating characteristics (ROC) curve for the suggested model for 60 epochs. The ROC curve has an AUC of 0.98 indicating high effectiveness in minimising both false positives and false negatives, indicating excellent overall performance.

**Table 6:** 5-fold cross validation results

Epochs of trained model	Test accuracy	Precision	Recall	F1 score	AUC
10	0.88	0.90	0.85	0.88	0.95
20	0.92	0.93	0.90	0.91	0.97
60	0.96	0.97	0.94	0.95	0.98

### Cross-validation on PTB-ECG dataset

Cross-validation of the pre-trained model was conducted on the PTB-ECG dataset, yielding promising results. The model was initially adapted to handle the variations in signal lengths present in the PTB ECG dataset. A resizing technique was applied to standardize all ECG signals to a consistent length, ensuring compatibility with the model. The dataset labels were transformed into a binary format for classification between “Myocardial infarction” and “Healthy control,” and the model architecture was adjusted by introducing additional dense layers to facilitate this binary classification.

After retraining the model with a batch size of 32 and monitoring performance using the validation dataset, the model was evaluated on the filtered and resized PTB ECG dataset. The results were promising, with the model achieving a test accuracy of 91.18%, demonstrating its potential for accurate classification of myocardial infarction and healthy controls. The training process involved resampling the signals to 500 Hz, normalizing them to a range of [-1, 1], and resizing the signals to a fixed length of 12,000 data points. To fine-tune the pre-trained model, the last few layers were unfrozen, and early stopping was implemented to prevent overfitting. After 5 epochs of training, the model achieved a training accuracy of 95.25% and a final test accuracy of 91.18%. These results are promising, indicating the model’s potential in achieving high performance on the PTB-ECG dataset, with the possibility of being further refined and applied to real-world clinical scenarios.

### Comparative evaluation against existing models

#### Comparison with existing models with PTB-XL dataset

Table 7 presents an examination of the comparative results achieved by the proposed method in contrast to prior works that use the PTB-XL dataset. The table highlights the performance of several models. In comparison, the proposed model, MI-CNN-DWT, incorporating DWT-based under-sampling, demonstrates notable performance with a testing accuracy of 96%, F1-score of 95%, and precision of 97%. This highlights the effectiveness of the proposed approach in achieving higher classification performance for MI and NORM.

**Table 7:** Comparison of existing models with PTB-XL

Ref No.	Number of classes	Classification between	Approach used	Highest accuracy achieved
Proposed model	2	Normal/MI	Discreate wavelet transform and convolutional neural networks	96% with 5-fold validation
[3]	2	Healthy/sick	CNN with entropy features	89%
[15]	5	NORM/MI/SSTC/CD/HYP	Visibility graph representation + Resnet/Inception	89.71% accuracy, 79.61% F-score, 3.46% AUC
[16]	5	NORM/MI/SSTC/CD/HYP	Beat segmentation/CNN + Attention. Bi-LSTM	88.85% mean accuracy 0.9216 Macro ROC-AUC 0.8057 F1 score

#### Comparison with existing models with PTB ECG diagnostic dataset

Table 8 shows the comparative results achieved by the proposed method in contrast to prior works that use the PTB ECG dataset. The proposed model, MI-CNN-DWT, incorporating DWT-based under-sampling, demonstrates notable performance with a cross-validation test accuracy of 91.18% highlighting the effectiveness of the proposed approach in achieving higher classification performance for MI and NORM.

**Table 8:** Comparison of existing models with PTB ECG dataset

Ref No.	Number of classes	Classification between	Approach used	Highest accuracy achieved
Proposed model	2	Normal/MI	Discreate wavelet transform and convolutional neural networks	91% with cross-validation
[13]	2	Normal/MI	CNN + LSTM	88.89%
[17]	2	Normal/abnormal	Symlet scaling filter and denoising/CNN (Alex Net), ELM	Accuracy 88.33%, sensitivity 89.47% and specificity 87.80%

#### Comparison with existing models with different dataset

Table 9 provides an overview of results from models using different datasets, such as the MIT-BIH Arrhythmia and PhysioNet/CINC challenge datasets, for similar classification tasks. Although the proposed MI-CNN-DWT model was not tested on these datasets, the table offers insights into the performance of existing models, providing context for comparing the MI-CNN-DWT model to other methods in ECG classification.

**Table 9:** Comparison with existing models with different dataset

Ref No.	Number of classes	Classification between	Approach used	Highest accuracy achieved	Dataset used
[6]	2	Abnormal/normal	CNN - LSTM	83.4%	MIT-BIH arrhythmia database
[7]	2	Myocardial Infraction/ Normal Myocardium	CNN-LSTM	85.1% for the left ventricular long-axis view and 83.2% for the short-axis view	Images taken with ultrasound equipment at Fujita health university hospital
[8]	2	Abonormal/normal	CNN	86%	Collected ECG data related to the study subject
[9]	17	Normal sinus rhythm, paced rhythm and 15 types of other arrhythmias	CNN	87.78%	MIT-BIH Arrhythmia database
[10]	2	Healthy/MI	CNN	87%	Physikalisch-Technische Bundesanstalt database
[11]	4	normal sinus rhythm, arrhythmic, other rhythm, noisy	Deep CNN and BiLSTM	89%	2017 PhysioNet/ CINC challenge dataset
[12]	4	normal sinus rhythm, arrhythmic, other rhythm, noisy	1D CNN combined with FCN layers	86%	2017 PhysioNet/ CINC challenge dataset
[14]	2	Normal/MI	Markov models and Gaussian mixture models	82.5%	Obtained from the Taoyuan Armed Forces General Hospital located in Taiwan

## Conclusion

In summary, this paper outlines an effective approach for classifying Electrocardiogram (ECG) signals, with a specific focus on distinguishing between ‘myocardial infarction’ (MI) and ‘normal’ cases. The best performing model, trained on filtered signals and evaluated using 5-fold validation, achieved an average accuracy of 96% with a precision of 97%. It effectively classified ECG signals and identified ‘MI’ (Myocardial Infarction) and ‘Normal’ cases. This model also exhibited an 94% recall, an F1 score of 95%, and an impressive AUC of 0.98, signifying strong ability to distinguish between MI and normal class.

Furthermore, the cross-validation accuracy on the PTB ECG dataset is a notable 91.18%, which highlights the model’s applicability to different datasets and its potential for broader clinical use. In conclusion, this research paper presents a robust ECG signal classification model, particularly adept at MI detection. This model’s architecture incorporates convolutional layers, Leaky-ReLU activation functions, dropout layers, and a meticulously designed learning rate schedule, making it a powerful tool for MI detection and cardiac condition diagnosis.

## Data Availability

The datasets generated during and/or analyzed during the current study are available in the following database • https://physionet.org/content/ptb-xl/1.0.3/ • https://physionet.org/content/ptbdb/1.0.0/.

## Abbreviations

MI: Myocardial Infarction
DWT: Discrete Wavelet Transform
CNN: Convolutional Neural Networks
CVDs: Cardiovascular diseases
ECGs: Electrocardiograms
CAD: Coronary artery disease
LSTM: Long Short-Term Memory
DCNN: Deep Convolutional Neural Network
BiLSTM: Bidirectional Long Short-Term Memory
EDN: Enhanced Deep Neural Network
HMMs: Hidden Markov models
GMMs: Gaussian mixture models
ReLU: Rectified Linear Unit
ROC: Receiver operating characteristics
AUC: Area Under ROC Curve
